# Successional changes in the chicken cecal microbiome during 42 days of growth are independent of organic acid feed additives

**DOI:** 10.1186/s12917-014-0282-8

**Published:** 2014-11-27

**Authors:** Brian B Oakley, R Jeff Buhr, Casey W Ritz, Brian H Kiepper, Mark E Berrang, Bruce S Seal, Nelson A Cox

**Affiliations:** Poultry Microbiological Safety Research Unit, USDA Agricultural Research Service, 950 College Station Road, Athens, GA 30605 USA; Western University of Health Sciences, College of Veterinary Medicine, 309 E. 2nd St, Pomona, CA 91766 USA; Poultry Science Department, University of Georgia, Athens, GA 30602 USA; Bacterial Epidemiology and Antibiotic Resistance Research Unit, USDA Agricultural Research Service, Richard B. Russell Agricultural Research Center, 950 College Station Road, Athens, GA 30605 USA

## Abstract

**Background:**

Poultry remains a major source of foodborne bacterial infections. A variety of additives with presumed anti-microbial and/or growth-promoting effects are commonly added to poultry feed during commercial grow-out, yet the effects of these additives on the gastrointestinal microbial community (the GI microbiome) as the bird matures remain largely unknown. Here we compared temporal changes in the cecal microbiome to the effects of formic acid, propionic acid, and medium-chain fatty acids (MCFA) added to feed and/or drinking water.

**Results:**

Cecal bacterial communities at day of hatch (n = 5 birds), 7d (n = 32), 21d (n = 27), and 42d (n = 36) post-hatch were surveyed using direct 454 sequencing of 16S rRNA gene amplicons from each bird in combination with cultivation-based recovery of a *Salmonella* Typhimurium marker strain and quantitative-PCR targeting *Clostridium perfringens*. Treatment effects on specific pathogens were generally non-significant. *S.* Typhimurium introduced by oral gavage at day of hatch was recovered by cultivation from nearly all birds sampled across treatments at 7d and 21d, but by 42d, *S.* Typhimurium was only recovered from ca. 25% of birds, regardless of treatment. Sequencing data also revealed non-significant treatment effects on genera containing known pathogens and on the cecal microbiome as a whole. In contrast, temporal changes in the cecal microbiome were dramatic, highly significant, and consistent across treatments. At 7d, the cecal community was dominated by three genera (*Flavonifractor*, *Pseudoflavonifractor*, and a *Lachnospiracea* sequence type) that accounted for more than half of sequences. By 21d post-hatch, a single genus (*Faecalibacterium*) accounted for 23-55% of sequences, and the number of *Clostridium* 16S rRNA gene copies detected by quantitative-PCR reached a maximum.

**Conclusions:**

Over the 42 d experiment, the cecal bacterial community changed significantly as measured by a variety of ecological metrics and increases in the complexity of co-occurrence networks. Management of poultry to improve animal health, nutrition, or food safety may need to consider the interactive effects of any treatments with the dramatic temporal shifts in the taxonomic composition of the cecal microbiome as described here.

**Electronic supplementary material:**

The online version of this article (doi:10.1186/s12917-014-0282-8) contains supplementary material, which is available to authorized users.

## Background

Foodborne pathogens reportedly accounted for 47 million episodes of illness and over 100,000 hospitalizations at an estimated cost of $77 billion in the United States in 2011 [1,2]. Foodborne illnesses are commonly associated with consumption of mishandled or improperly cooked poultry despite several decades of basic and applied food safety research. Interventions designed to reduce the incidence of poultry-associated foodborne illness are generally targeted either to the later stages of poultry processing such as chlorinated chill tanks commonly used in the U.S [3,4], or on-farm interventions which seek to reduce pathogen loads at various stages of the production process prior to processing [5]. Feed additives that can modulate the gastrointestinal microbial community (the GI microbiome) have been the subject of intense and increasing interest following the 2006 European Union ban on prophylactic antibiotics added to feed as growth promoters [6] and calls for similar regulation in the U.S. [7-9].

To evaluate the efficacy and utility of alternative antimicrobial feed additives, two main parameters need to be evaluated: 1) the effect of the additive on the pathogen(s) of interest, and 2) the effects of the additive on the GI microbiome of the host. The importance of GI microbial communities for the health and nutrition of the host organism is now well established [10-14], and removing antibiotics from feed (as proposed by recent FDA guidance for industry) has previously been shown to induce various changes within the chicken GI microbiome [15-18]. Developing acceptable alternatives to antibiotics will thus require assessing their effect on specific pathogens and the GI microbiome.

To date, a number of studies have examined the effects of various alternative antimicrobial feed additives on GI microbial communities of poultry [15,19-25], but only recently have a few studies [26-29] utilized the power of modern high-throughput sequencing (HTS) to provide a comprehensive taxonomic census and fully assess the effects of treatments on the GI microbiome. Methods commonly used in the past have some important shortcomings, including extreme taxonomic bias (cultivation-based approaches), low taxonomic resolution (DGGE, T-RFLP), or inadequate depth of sampling (Sanger-sequenced clone libraries). Another important aspect of any evaluation of a feed additive is determining how any treatment effects interact with natural successional changes in the GI microbiome. Dramatic changes in community composition and function have been shown to occur naturally as birds mature [18,30-33], although most of these previous studies share the same methodological limitations discussed above and thus are in need of revisiting with modern methods.

In this work we combine 454 pyrosequencing of broad-range 16S rRNA gene amplicons, quantitative-PCR, and cultivation-based recovery of a pathogenic marker strain to document the successional development and effects of feed additives on the cecal microbiome and specific pathogens. By sequencing a population of amplicons to exhaustion, HTS performs a comprehensive census free of cultivation bias; these taxonomic data are necessary to improve understanding of the community structure of the poultry cecal microbiome and how it changes as birds mature to market age. The work presented here had two main objectives: 1) determine the relative effects of organic acid feed additives and successional changes in the poultry cecal microbiome at the community level using HTS and on the pathogens *Salmonella* and *Clostridium* using cultivation and qPCR respectively, and 2) provide a comprehensive data set of the taxonomic composition of the cecal microbiome in broilers as they grow to market age.

## Results and discussion

### Effects of treatments versus time on cecal microbiome

**Figure 1:**
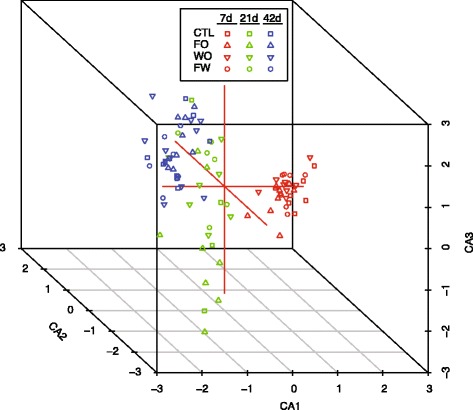
**Clustering of the cecal microbiome by treatment and time.** Clustering was performed by canonical correspondence analysis as described in the text. Each point represents a single bird with sequences clustered independent of taxonomic assignments according to operational taxonomic units (OTUs) defined at a 97% similarity cutoff as described in the text. Data from day-of-hatch birds group off of the axes and are excluded for clarity. Clustering based on classification of sequences to the genus or species level gave equivalent results. Treatment designations are Ctl, control; FO, feed-only; WO, water-only; and FW, feed and water as described in the text.

Temporal changes in the cecal microbiome were dramatic, highly significant, and consistent across treatments. Clustering of the cecal microbiome from each bird at the OTU level using CCA as described in the methods showed clear groupings by time that were much stronger than any treatment effect (Figure 1). To explicitly test the relative effects of time versus experimental treatments on the cecal microbiome, permutational MANOVA was used as described in the methods. The effect of time was highly significant (p < 0.0001) whereas experimental treatment effects were non-significant (Table 1). Clustering and hypothesis testing using taxonomic classifications of sequences to the genus level gave with the RDP classifier or Silva database equivalent results.

**Table 1:** **Results of permutational MANOVA conducted with the adonis function in R**

	**Degrees of freedom**	**Sum of squares**	**Mean squares**	**F. model**	**R** ^**2**^	**Pr** **(>F)**
Time	1	3.457	3.457	8.986	0.0912	**0.0001**
Treatment	3	1.137	0.379	0.985	0.0300	0.4935
Time:Treatment	3	1.019	0.340	0.883	0.0269	0.8455
Residuals	84	32.310	0.385		0.8520	
Total	91	37.922			1.0000	

Comparisons were made using OTU-level classification of the sequencing reads for each bird and 10,000 permutations. Only the effect of time on community composition was significant (p < 0.0001). Taxonomic classifications of sequences against the RDP or Silva databases gave equivalent results.

**Figure 2:**
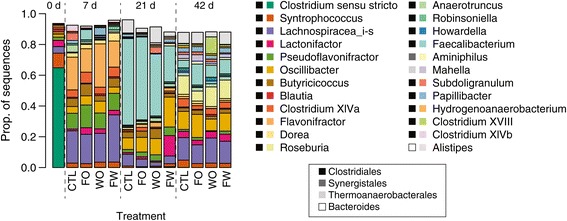
**Relative abundance at the genus level for sequences by treatment and time with taxonomic classifications performed with the RDP classifier as described in the text.** Only sequences with a total relative abundance greater than 5% are shown. For day-of-hatch birds and each subsequent time point (7d, 21d, and 42 d post-hatch), the relative proportions are shown for each treatment. Day-of-hatch birds were proportionally high in *Clostridium* but low quantitatively as shown in Figure 3. Treatment designations are Ctl, control; FO, feed-only; WO, water-only; and FW, feed and water as described in the text.

To document the composition of the cecal microbiota and examine specific changes through time and by treatment, sequences were classified taxonomically. At 7d, the cecal community was dominated by three genera (*Flavonifractor*, *Pseudoflavonifractor*, and *Lachnospiracea incertae sedis*; the latter sequences mostly classified as *Blautia* or *Ruminococcus* by usearch against the Silva database) that accounted for more than half of sequences (Figure 2). These three groups all belong to the Clostridiales with *Flavonifractor* and *Pseudoflavonifractor* quite closely related phylogenetically. *Blautia* has recently been identifed as a ubiquitious (though low abundance) taxon present in humans and various animals [34], and members of the Clostridiales are well known for their conversions of complex polysaccharides to short chain fatty acids such as butyrate that have significant positive growth effects [35,36].

By 21d post-hatch, a single genus (*Faecalibacterium*) accounted for 23-55% of sequences (Figure 2). *Faecalibacterium prausnitzii* has been shown to have anti-inflammatory properties and an inverse correlation with severity and recurrence of colitis in humans and murine models [37]. Whether or not members of the genus *Faecalibacterium* have similar roles in chickens remains an interesting question. By 42d, *Faecalibacterium* sequences were recovered at approximately equal proportions to *Roseburia*, a saccharolytic, butyrate-producing bacterium [38]. Also relatively abundant at 42 d were sequences classified as *Lachnospiracea incertae sedis*, and *Oscillibacter*, previously encountered in chickens [39] and with some members known to produce short chain fatty acids [40,41]. These data are consistent with previous results identifying various members of the poultry GI microbiome [15,19-25,42], but by exhaustive sequencing with modern methods from a fairly large number of birds, also provide important new information regarding the generic composition of the chicken cecal microbial community and how it changes through time. Proper understanding and management of temporal changes in the GI microbiome will be important for maintaining bird health and improving productivity.

### Effects of treatments versus time on specific pathogens

Treatment effects on specific pathogens were generally non-significant. The marker strain of S. *typhimurium* was recovered from nearly all birds sampled at 7d and 21d, regardless of treatment, and by 42d, few differences were observed across the treatments with the marker strain recovered from ca. ¼ of treated and untreated birds (Table 2). Importantly, from 21 d to 42 d the proportion of birds from which *Salmonella* was recovered across all treatments decreased from a mean of 94% to 26% (Table 2).

**Table 2:** **Proportion of birds with positive culture tests for marker strain of** ***Salmonella*** **Typhimurium recovered from cecal samples**

**Treatment**	**7d**	**21d**	**42d**
Ctl	8/8	3/4	7/20
WO	8/8	4/4	5/20
FO	8/8	4/4	4/20
FW	8/8	4/4	5/20
Total	100%	94%	26%

Treatment designations are Ctl, control; FO, feed-only; WO, water-only; and FW, feed and water. Cultivation media and methods are described in detail in the text.

**Figure 3:**
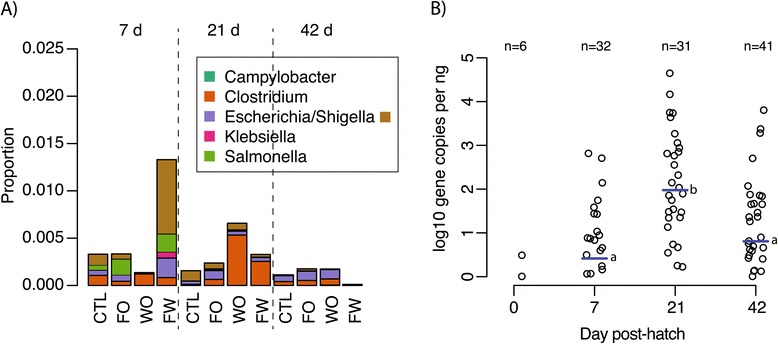
**Changes in relative abundance of putative pathogens by treatment and time. A)** For each time point (7d, 21d, and 42 d post-hatch), the relative proportions are shown for each of the four treatments. Putative pathogens were defined using the intersection of independent taxonomic classifications with the RDP classifier and the Silva database as described in the methods. Sequences classified as Escherichia or Shigella by Silva are shown separately but not distinguished by RDP. Treatment designations are Ctl, control; FO, feed-only; WO, water-only; and FW, feed and water as described in the text. Note scale of Y axis. **B)** Number of gene copies of *Clostridium* as determined by quantitative-PCR for each time point. Treatments for each time point are grouped due to the non-significant effect of treatment as shown in Table 1. Quantitative loads of *Clostridium* were significantly higher at 21 d than 7d or 42 (p < 0.0001, one-sided t-tests),

Sequencing data also demonstrated small treatment effects on taxonomic groups containing known pathogens (Figure 3). Consistent with the cultivation data, *Salmonella* sequences decreased in relative abundance with time and were almost entirely absent by 21d (Figure 3A). Sequences classified as *Clostridium* increased to a maximum of 0.5% at 21d, subsequently decreasing in relative abundance at 42d. (Figure 3A). In general, taxa considered as putative pathogens (*Campylobacter, Clostridium*, *Escherichia/Shigella*, *Klebsiella*, and *Salmonella*) were a minor component of the community (<1.5% total relative abundance). Quantitative-PCR for the *Clostridium* clade containing the *C. perfringens* subgroup was qualitatively consistent with the sequencing data and showed a significant increase in the abundance of this group from day of hatch to 21d post-hatch, followed by a significant decline by 42d to the same levels at 7d (Figure 3B).

Although the main purpose of the work presented here was to monitor the cecal microbial community as a whole, comparing the sequencing and qPCR data for *Clostridium* helps to validate the use of sequencing, even for relatively low abundance taxa. Assumptions of a mean genome size of 3.6 Mbp [43] and bacterial cell densities in the chicken cecum of 10^10^-10^11^ cells g^−1^ [44], gives approximately 4 × 10^4^ ng bacterial DNA g^−1^ of cecal contents. Further, applying an extraction efficiency assumption of 14% [45] to the qPCR data at 21d post-hatch where ca. 100 *C. perfringens* group rRNA gene copies were observed ng^−1^ of DNA, gives 2.8 × 10^6^ cells g^−1^ of cecal contents, or 0.028% of a total bacterial community of 10^10^ cells. From the sequencing data, the proportion of *Clostridium* sequences at 21d post-hatch was approximately an order of magnitude greater (0.216%), which is roughly in line particularly as the sequence data were classified at the genus level.

Clostridia are abundant numerically and proportionally in the chicken GI microbiome, particularly in the ceca [16,46]. *Clostridium islandicum* and other members of the *Clostridium* cluster XIV are associated with cellulytic activity [47] and feed conversion [48], while other Clostridia such as *C. perfringens* are veterinary pathogens causing enteric diseases in both domestic and wild animals, gas gangrene (clostridial myonecrosis), necrotic enteritis, and gastrointestinal infections in humans [49-51]. As the mechanisms for colonization of the avian intestinal tract and the factors involved in toxin production remain largely unknown, few tools and strategies are currently available for prevention and control of *C. perfringens* in poultry. Vaccination against this pathogen and the use of probiotic or prebiotic products has been suggested, but are not available for practical use in the field [51]. Although no disease was overtly observed during our experiment, low levels of *C. perfringens* were detected in the ceca of treated and untreated chickens.

**Figure 4:**
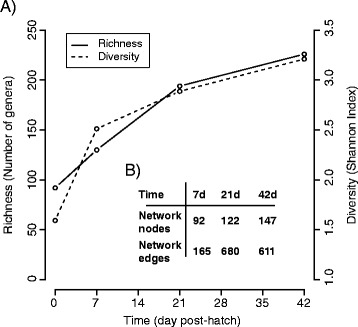
**Taxonomic richness and diversity of the cecal microbiome at the genus level through time. A)** Richness and diversity statistics calculated at the species and OTU-level showed essentially similar patterns through time. **B)** Network complexity of the cecal microbiome through time as measured by the numbers of nodes and edges in network. Nodes represent genera with significant network connections to other genera and edges represent the total number of significant networks connections calculated as described in the text.

Through the course of the experiment, the cecal community became more taxonomically rich and diverse. The number of genera more than doubled to >200 at 42 d and diversity increased similarly (Figure 4). Network analysis performed on the cecal microbiome at each time point also showed an increase in complexity with the number of nodes (taxa with significant co-occurrence patterns with other taxa), increasing through time (92, 122, and 147 nodes at 7 d, 21d and 42 d respectively; Figure 4). Previously, increases in taxonomic richness as birds mature has been inferred from the number of bands on DGGE gels [30,32], TRFLP fingerprints, and sequencing [33]. Metagenomic approaches have provided important insights into the poultry cecal microbiome [52] and the effects of antimicrobials [18], but 16S rRNA-based taxonomic profiling provides the most relevant information for food-safety regulations and the development of probiotic or other alternatives to antibiotics, such as phage-lytic enzymes.

## Conclusions

Although organic acids as feed additives have been proposed as a management strategy in various formulations to combat pathogens in poultry [53-58], we found little effect of the treatments tested here on specific pathogens or the cecal microbiome in general. We did observe dramatic changes in the cecal microbiome through time, consistent with earlier work [33], and for the first time, detail these changes taxonomically using high-throughput sequencing. During the 42 d of the experiment, the cecal microbiome became significantly more taxon-rich and diverse according to a variety of ecological metrics and increases in network complexity. The temporal dynamics of the poultry GI microbiome need to be considered in the proper management of poultry for productivity, animal health, and food safety.

## Methods

### *Experimental procedures*

A total of 480 male chicks (Ross × Cobb broilers) in a feeding trial were given one of four experimental feeding treatments beginning at day of hatch: 1) a solution of formic acid (CH_2_O_2_; 340 ppm final concentration), propionic acid (C_3_H_6_O_2_; 250 ppm), ammonium formate (NH_4_HCO_2_; 200 ppm medium-chain fatty acids (MCFA; 100 ppm), an emulsifier (50 ppm), and propylene glycol (1 ppm) added to the drinking water (n = 120), 2) propionic acid (480 ppm) and MCFA (1520 ppm) added to the feed (n = 120), 3) a combination of treated feed and water at the same concentrations (n = 120), or 4) control birds receiving a standard nonmedicated corn-soybean diet (n = 120). These treatments are hereafter referred to according to the treated component as water-only (WO), feed-only (FO), feed and water (FW), or control (CTL). Day of hatch chicks were placed on clean pine shavings in ca. 1 m x 3 m floor pens with feed and water (via nipple drinker lines) provided ad libitum. Each of the four treatments included four replicate pens for a total of 16 pens, each containing 30 birds. Within each pen, two birds were orally inoculated as ‘seeder birds’ with 0.1 mL of a 10^7^ cells ml^−1^ suspension of a nalidixic acid resistant strain of *Salmonella* Typhimurium. At each of four time points (0, 7, 21, and 42 d post-hatch), ceca were collected for cultivation and DNA extractions as previously described [59]. Sample sizes and details are shown in Table 3. Ethical approval of animal work was granted under University of Georgia animal use permit A2012 02-002-Y2-A0.

**Table 3:** **Number of birds sampled for quantitative**-**PCR and 454 pyrosequencing by treatment and time**

**Treatment**	**0d**		**7d**		**21d**		**42d**		**Subtotal**	
	**qPCR**	**454**	**qPCR**	**454**	**qPCR**	**454**	**qPCR**	**454**	**qPCR**	**454**
Ctl	6	6	8	8	7	4	10	10	31	22
WO	n/a	n/a	8	8	8	8	11	10	27	26
FO	n/a	n/a	8	8	8	8	11	9	27	25
FW	n/a	n/a	8	8	8	7	9	7	25	22
Subtotal	6	6	32	32	31	27	41	36	110	95

Treatment designations are Ctl, control; FO, feed-only; WO, water-only; and FW, feed and water.

### Quantitative-PCR, 454 sequencing and data analysis

Quantitative-PCR assays for the *C. perfringens* group were performed as previously described [46] with forward (5′-ATGCAAGTCGAGCGAKG-3′) and reverse (5′-TATGCGGTATTAATCTYCCTTT-3′) primers from [60] and SYBR Green chemistry (ABI, Carslbad, CA).

PCR and 454 pyrosequencing of the V1-V3 regions of 16S rRNA genes were performed using tagged amplicon methods as previously described [46,61]. Briefly, sequences were de-multiplexed and preprocessed with the Galaxy toolkit [62] and our own custom tools [63]; additional quality controls per recent recommendations and standard protocols [64] were completed using Perl and Bioperl scripts to trim pyrosequencing tag sequences, screen for presence of the forward PCR primer sequence, and remove sequences with any ambiguous base calls. Based on expected amplicon sizes and frequency distributions of sequence lengths in v108 of the Silva reference database, sequences were further limited to a range of 325–425 bp. Putative chimeric sequences were identified with usearch [65] and ChimeraSlayer in mothur [66]. After these screening steps, the following number of sequences per treatment group were used for analysis: CTL 7d 31280; FO 7d 31174, FW 7d 33990, WO 7d 33844, CTL 21d 18902, FO 21d 25491, FW 21d 23114, WO 21d 32309, CTL 42d 38770, FO 42d 32167, FW 42d 25578, WO 42d 34168. Rarefaction curves are shown in Additional file 1: Figure S1.

Taxonomic classification of sequences was performed with the RDP naïve Bayesian classifier [67] v2.6 and the EMBL taxonomy from v115 of the Silva project curated seed database using usearch with the global alignment option [65]. To assess phylotype richness and diversity independent of taxonomic classifications, sequences which passed all the screens described above were grouped into similarity clusters (operational taxonomic units; OTUs), using similarity cutoffs of 90%, 95%, and 97% with uclust [65]. The output from usearch provided the inputs for our own customized analysis pipeline to parse the clustering results and produce graphical and statistical summaries of the data for the desired sampling units using perl and R [68] as previously described [61,63]. Clustering of communities was performed using the CCA function of the vegan package [69] in R based on OTU and taxonomic classifications. The relative effects of time (number of days post-hatch) versus experimental treatment (and their interactive effects) on cecal microbial communities was determined by a permutational multivariate analysis of variance (MANOVA) using the adonis function of the vegan package in R. Briefly, OTU or taxonomic classifications of sequences from each bird are used to partition sums of squared deviations from centroids in a distance matrix to determine how variation is explained by experimental treatments (feed additives and/or sampling time in our case), or uncontrolled covariates [70].

Network analysis was conducted as previously described [46] using normalized OTU tables at various levels of clustering and removing OTUs or taxa represented by fewer than five sequences or <0.5% total relative abundance across all samples. Spearman correlation coefficients of 0.7 and p-values of 0.001 were required to establish valid co-occurrence among OTUs. Network analysis was performed in R with the igraph package and visualized with the program Gephi.

Sequence data have been deposited in GenBank with accession numbers SAMN03161778-SAMN03161871 associated with BioProject ID 263495.

## Additional file

Additional file 1: Figure S1. Rarefaction curves for genus (A), species (B) and 3 percent OTU (operational taxonomic unit) classifications (C). Note differences in Y axis scaling.
