# Supplementary material for: Successional changes in the chicken cecal microbiome during 42 days of growth are independent of organic acid feed additives
Source: BMC Vet Res. 2014 Nov 27;10:282. doi: 10.1186/s12917-014-0282-8 (PMC4251860; doi:10.1186/s12917-014-0282-8)
Supplement: Additional file 1: Figure S1. — Rarefaction curves for genus (A), species (B) and 3 percent OTU (operational taxonomic unit) classifications (C). Note differences in Y axis scaling. [file 12917_2014_282_MOESM1_ESM.pdf]

A)

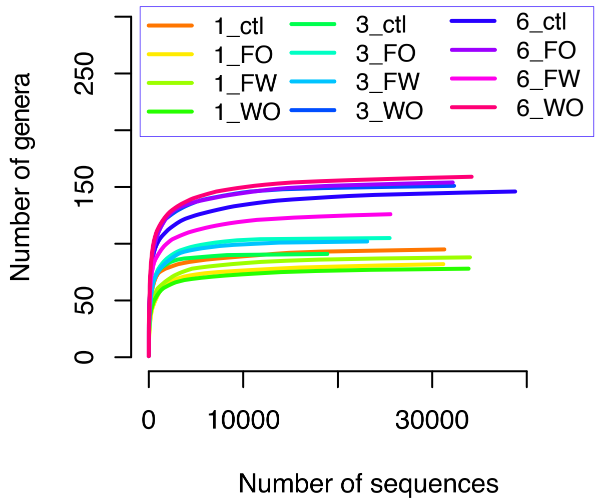

B)

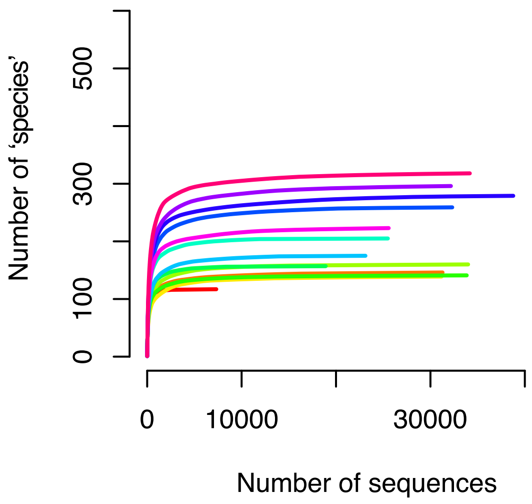

C)

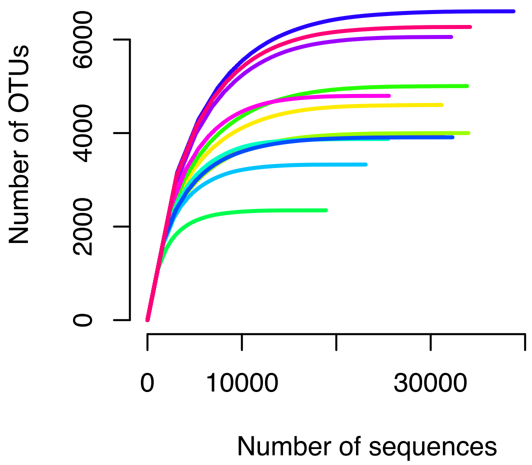

Supplementary Figure 1. Rarefaction curves for genus (A), species (B), and 3 percent OTU (operational taxonomic unit) classifications (C). Note differences in Y axis scaling.
